# Coronavirus (SARS-CoV-2) and Mortality Rate in India: The Winning Edge

**DOI:** 10.3389/fpubh.2020.00397

**Published:** 2020-07-21

**Authors:** Gyaneshwer Chaubey

**Affiliations:** Cytogenetics Laboratory, Department of Zoology, Banaras Hindu University, Varanasi, India

**Keywords:** COVID-19, India, endogamy, disease susceptibility, ACE2, herd immunity

The Covid-19 outbreak is due to a virus which emerged in China at the end of December 2019, and is now widespread in more than 200 countries worldwide (1). Several researches have highlighted that it was introduced to humans from bats (2, 3). The infection spreads like a chain reaction as one infected person passes this virus to two or three others, who then continue to spread it in a similar manner (Figure 1). The number of people infected in a region from a single person is estimated as R_0_ (R zero). R_0_ is the rate at which new infections stem from a single case (4). R_0_ < 1 indicates the reduction of cases, whereas R_0_ > 1 suggests that the number of cases are increasing. The global R_0_ value for Covid-19 is estimated to range between 3 and 5, which is twice as fast as SARS (Severe Acute Respiratory Syndrome) (5). This is why the spread of Covid-19 is so rapid, and why the number of infected people double every 5–10 days (Coronavirus in India). The socioeconomic impact of Covid-19 is disruptive, and the whole world is looking forward to the end of this crisis. Similar to other countries, its transmission among the Indian population is evident. But the major question is its fate in India, as India makes up one-fifth of the world's population. The recent report makes India's fate more vulnerable, by estimating that the total number of reported cases are 10-fold less than the total number of infected people (6). Thus, such complexity makes India one of the most monitored countries during this pandemic.

**Figure 1:**
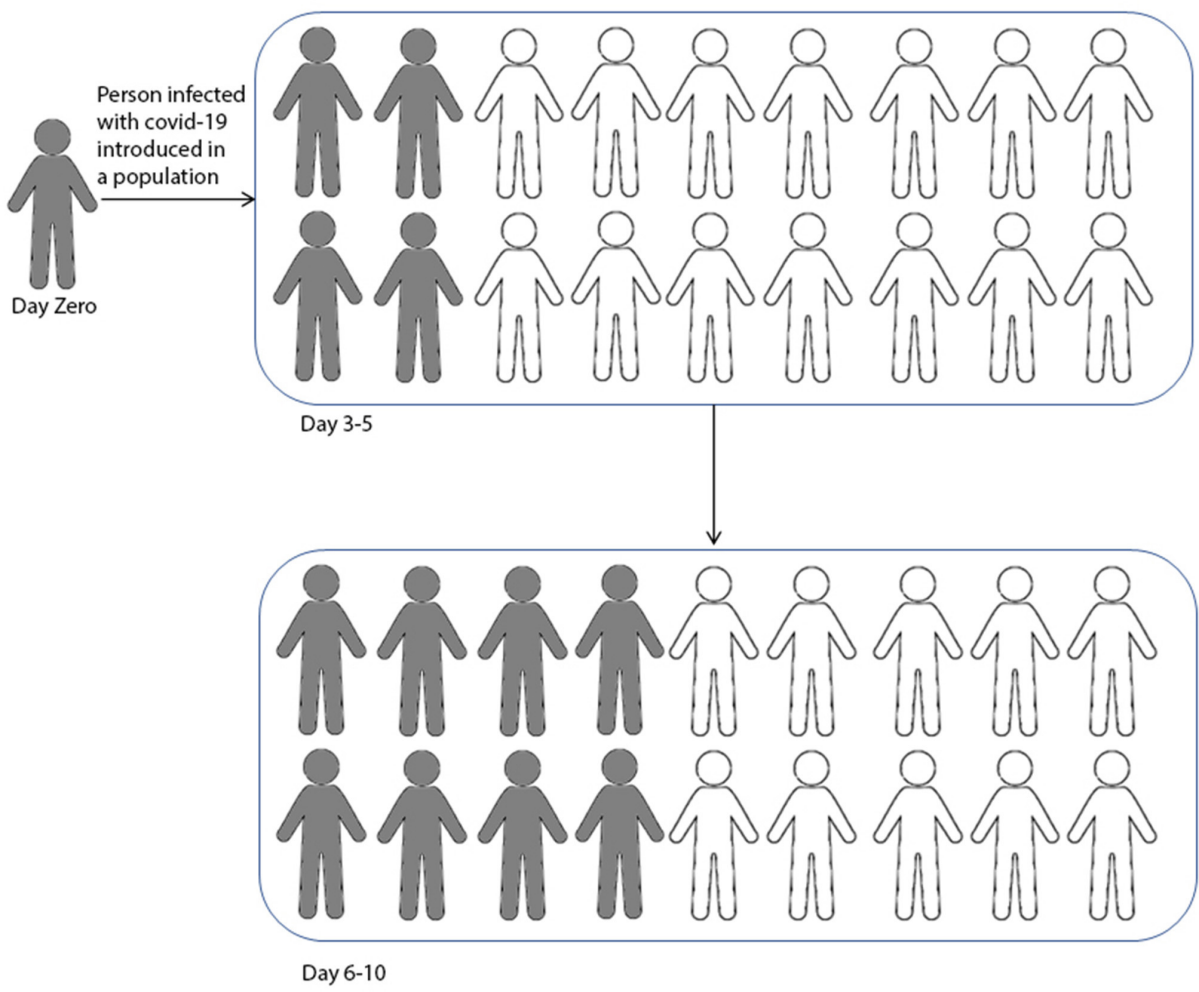
The graphical representation of Covid-19 infection in a natural population. The viral infection spread from peer-to-peer like a chain reaction. This figure is only a graphical representation of the spread, without considering whether someone in the population has recovered or died.

Contrary to what one would expect, the emerging data suggests that at the front of mortality, the situation in India turned out not to be as bad as in some European nations and the USA. However, given the fact that India's emergency services are limited, it is likely to be more vulnerable to this pandemic. Keeping this in mind, the Indian government has introduced unprecedented measures (including the stringent and early nation-wide lockdown from March 22), to stop the spread of Covid-19. Wherever a high number of cases are found, it is considered as a hotspot. The locality is immediately sealed to stop the spread of the virus. Further, in lockdown 3.0, the country was divided in to green, orange, and red zones, based on severity and the number of cases (MOHFW-GoI)^1^. However, we are not sure how long this measure should be implemented and what the chances of resurgence will be when these restrictions are relaxed after a few weeks. Equally at the research front, scientists from all over the world are trying to find a way to exit from this crisis. More than 20,000 research papers (doubling every 20 days) on this topic itself suggests its seriousness (7). Indeed, social distancing and other governmental measures would reduce the virus's ability to sweep through the population, but unless, or until a vaccine is discovered, what measures can India rely upon to control the spread of the virus?

The one and most prominent way is to obtain “herd immunity” (8). When a population is exposed to any infectious disease, many of its inhabitants gain immunity in a short period of time. When ~70% of individuals in the population become immune, this facilitates herd immunity. This barrier of immunity blocks the virus from taking hold and infecting others. Immunity may be sustained for almost a year, and such a time period can buy us time to develop a vaccine. Moreover, immune people can volunteer for the healthcare services and other necessary activities without any sophisticated protective gear. This seems easy to implement, but when looking fatalities figures for the coronavirus, we cannot put the life of ~3% (or even more) people on the line (Figure 2A). Therefore, thinking of obtaining rapid herd immunity in this particular case, is an autophagy for a nation like India. A recent example is the UK, which had initiated this method in the beginning as a measure, but seeing the severe impact of the virus, abandoned this plan (9, 10).

**Figure 2:**
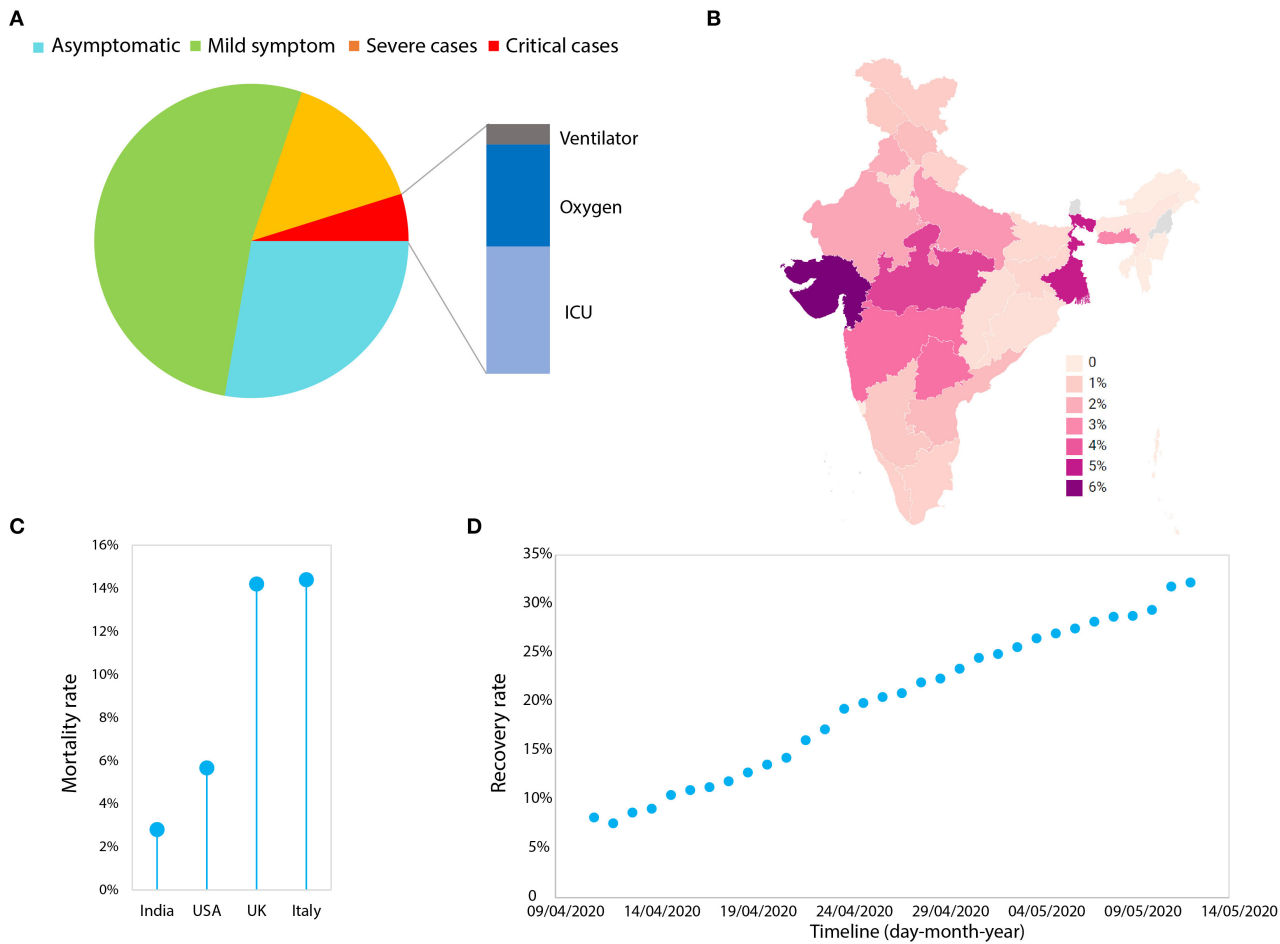
The analysis of Covid-19 from India. **(A)** The frequency (%) of infected people with various levels of symptoms. The data was obtained from (https://www.mohfw.gov.in/). **(B)** The statewide mortality rate (number of people deceased/total number of reported cases) of the infected people. The data was obtained from the https://www.covid19india.org/ and plotted through (https://app.datawrapper.de/). **(C)** The mortality rate of India in comparison with the USA, the UK, and Italy. **(D)** The plot of day-by-day recovery rates (number of people recovered/total number of reported cases) in India (last updated: 12th May 2020). The data was obtained from (https://www.mohfw.gov.in/).

Accumulating research in other regions of the world are suggesting the effect of high temperatures and humidity on Covid-19 (11–13). However, so far, none of the dedicated studies in India have been performed on the association of temperature or humidity on the spread of Covid-19. Therefore, the relevance of temperature on the spread of Covid-19 in India is not known.

Recent research suggests that the coronavirus receptor of human *ACE2* plays a pivotal role in disease predisposition, therefore, certain polymorphisms in this gene may affect the susceptibility of a population (14–21). As an expert on Human Evolutionary Genetics, it is necessary to reiterate that, in India, modern humans have been living for at least 50,000 to 70,000 years and have experienced various kinds of pathogen pressures (22–25). A large number of genetic and archaeological studies are consistent with a largely local emergence of South Asian ancestry with minor [and in some cases relatively higher e.g., Tibeto-Burmans (26), Austroasiatics (27), and some Northwest Indian populations (28)], ancestry contributions from East and West Eurasians respectively (29–31). Therefore, these long term geographic and genetic isolations, might have certainly helped us to modify our genetic landscape against various kinds of pathogens (22, 25, 32–34). Moreover, the high level of endogamy practices among caste and tribal populations (29, 35) has created a unique genetic profile, and thus, likely variations in *ACE2*. Therefore, it is likely that many of the endogamous populations might have developed a varied degree of susceptibility responses against this virus.

In fact, Cao et al. (15) have looked at the binding sites of the S protein of coronavirus but did not find any variation among 1,000 genome populations. However, keeping in mind that 1,000 genome South Asian samples do not capture the complete South Asian diversity (36), one should look at these variations as well as whole *ACE2* variations in the large number of South Asian ethnic groups. Moreover, the greater role of *ACE2* in disease manifestation is evident in our recent study, which showed that the major South Asian *ACE2* haplotypes are identity by descent (IBD) of East Eurasians rather than West Eurasian (19), which is possibly one of the reasons for the low mortality among the Indian population. Therefore, studying the detailed *ACE2* variations among diverse Indian populations would be worthwhile to understand human susceptibility to Covid-19.

A study done by the Chinese Centre for Disease Control and Prevention on 72,314 cases suggested the presence of 1% asymptomatic individuals (Chinese Center for Disease Control and Prevention)^2^. Asymptomatic individuals are people who have been diagnosed to have a positive viral load but lack any characteristic symptoms including fever, dry cough, and fatigue etc. Recent data in India has identified 28% asymptomatic people—which is alarming (Figure 2A). Notably, ~65% of the Indian population is under the age of 35 years, thus the number of asymptomatic people would likely be much higher than reported (6). Therefore, it is highly alarming and brings to the forefront the question—how can one stop infections that are spread by asymptomatic people?

To identify asymptomatic people, two important dependent measures can be applied. First is mass antigen/antibody testing, and second is to look at their *ACE2* variations. In order to investigate the real spread estimation (6), as well as disease spread due to asymptomatic people, the government could initiate, first, mass antigen/antibody testing in containment zones, and second, to see if these people have certain *ACE2* polymorphisms in common. In a positive sense, this would not only help in identifying some of the asymptomatic ethnic groups, but could also help the government in substantially reducing infections (37, 38). Additionally, since the comorbidity is consistent all around the globe (39, 40), serious public awareness is required for those at high risk, to reduce the mortality. The statistics of high risk people in India with diseases such as Diabetes-2 (80M) and Cardiovascular diseases (53M) are daunting, and may require urgent attention (41, 42).

The first nation-wide lockdown of 21 days and further lockdowns 2.0, 3.0, and 4.0, was certainly helpful in restricting asymptomatic people. The governmental measures applied so far have substantially reduced the infection rate R_0_ (<1.5) with a doubling rate of cases (i.e., cases double every 15 days) for India [MOHFW-GoI^1^; (43)]. The data available to date suggest a mean death rate of 2.8% for India, which includes 86% comorbidity cases (Figure 2A). Many of the states are performing well except those states such as West Bengal and Gujarat, where death rates are closer to the USA i.e., >5% (Figures 2B,C). In fact, the recovery rate for India has increased from 7.6 to 32% in a month (Figure 2D). Additionally it is highly evident that mortality is extremely low compared to western nations (per 100,000 population; 0.2 for India, 26.6 for the USA and 52.1 for the UK) (Figures 2B,C), keeping in mind the low healthcare index 154/195 ranking of India (44). The lockdown measures, public awareness campaigns, and social distancing would have all likely contributed to this low mortality rate.

The growing number of cases in India suggests that the peak of infection has not yet occurred. However, if we look closely, 1/3rd of the cases have been accumulating in two cities, Mumbai and Delhi, and ½ of the cases have occurred in four cities (Mumbai, Delhi, Chennai, and Ahmedabad). If a similar situation occurs in another few densely populated cities, things may be not as under control as we are witnessing today. Therefore, each and every region of India has to develop their own prevention models, considering our past experiences in pandemics, literacy, and healthcare availability (45–47).

It is therefore in India's best interest to continue the use of standard protective measures (48, 49), to conduct systematic epidemiological studies in different real time situations, to test various ethnic groups for *ACE2* variations, to perform mass antigen/antibody testing in containment zones, to identify those who are immune, and to continue the lockdown until R_0_ reduces to <1. This will provide massive success in this current health crisis. Collectively, it is likely that with India's diverse ethnic groups and governmental measures, it will not be easy for Covid-19 to cause a large number of casualties.

## Author Contributions

The author confirms being the sole contributor of this work and has approved it for publication.

## Conflict of Interest

The author declares that the research was conducted in the absence of any commercial or financial relationships that could be construed as a potential conflict of interest.

## Footnotes

^1^Available at: mohfw.gov.in.

^2^http://www.chinacdc.cn/en/COVID19/ (accessed March 29, 2020).
